# Immunosenescence in the nursing home elderly

**DOI:** 10.1186/1471-2318-14-50

**Published:** 2014-04-17

**Authors:** Jennie Johnstone, Jamie Millar, Alina Lelic, Chris P Verschoor, Stephen D Walter, Philip J Devereaux, Jonathan Bramson, Mark Loeb

**Affiliations:** 1 Department of Clinical Epidemiology and Biostatistics, McMaster University, Hamilton, ON L8S 4 K1, Canada; 2 McMaster Immunology Research Centre, McMaster University, Hamilton, ON L8S 4 K1, Canada; 3 Department Pathology and Molecular Medicine, McMaster University, Hamilton, ON L8S 4 K1, Canada; 4 Department of Medicine, McMaster University, Hamilton, ON L8S 4 K1, Canada; 5 Institute for Infectious Disease Research, McMaster University, Hamilton, ON L8S 4 K1, Canada; 6 McMaster University, MDCL 3200, 1200 Main St W, Hamilton, Ontario L8N 3Z5, Canada; 7 McMaster University, MDCL 3203, 1200 Main St W, Hamilton, Ontario L8N 3Z5, Canada

**Keywords:** Immunosenescence, Aging, Immune phenotypes, Nursing home elderly

## Abstract

**Background:**

To describe T-cell and natural killer (NK) cell phenotypes within nursing home elderly.

**Methods:**

Nursing home elderly were recruited from four nursing homes in Hamilton, Ontario between September 2010 and December 2011. Healthy adults were recruited from McMaster University between September 2011 and December 2011. Nursing home elderly ≥65 years were eligible; those on immunosuppressive medications were excluded. Healthy adults ≥18-64 years were eligible. CD8+ and CD4+ T-cells% and their subsets, T-regs% and NK cell subset% were compared between the nursing home elderly and healthy adults.

**Results:**

262 nursing home elderly were enrolled; median age 87 years and 81% were female. 16 healthy adults were enrolled; median age 31 and 50% were female. There was no significant difference between CD8+ T-cell% in nursing home and healthy adults (median 17.1 versus 18.0, p = 0.56), however there were fewer naïve CD8 + T-cell% (median 0.9 versus 5.2, p < 0.001), more terminally differentiated CD8 + T-cell% (median 7.3 versus 4.1, p = 0.004) and more senescent T-cell% (median 5.3 versus 3.1, p = 0.04) in the nursing home elderly. There were more CD4+ T-cell% in the nursing home elderly compared to healthy adults (median 45.5 versus 37.1, p = 0.001). Nursing home elderly had a higher CD4+/CD8+ ratio than healthy adults (2.6 versus 1.9, p = 0.048), higher T-reg% (median 1.8 versus 0.8, p < 0.001) and increased mature NK cell% (median 12.1 versus 5.4, p = 0.001) compared to healthy adults.

**Conclusion:**

Differences in naïve CD8+ T-cells, terminally differentiated and senescent CD8+ T-cells, T-regs and NK cell subsets were similar to studies involving community dwelling elderly. In contrast, the CD4+/CD8+ ratio was higher in nursing home elderly.

## Background

Dysfunctional changes to the immune system that arise with age are termed immunosenescence [1]. Although all components of the immune system are affected by aging, changes to T-cells are by far the most pronounced [1]. Changes include a reduction in the number of naïve T-cells due to thymic involution, and an increase in CD8+ memory T-cells subsets including an accumulation of terminally differentiated memory T-cells (CD8 + CD45 + CCR7-) [2] and senescent cells (CD8 + CD28-) [3,4]. These changes have led to the identification of immune biomarkers; senescent T-cells were predictive of influenza vaccine failure in two community dwelling elderly cohorts [5,6] and an immune risk profile characterized by a CD4+/CD8+ ratio less than 1 (due to the expansion of the CD8+ T-cell pool relative to CD4+ T-cell pool) was predictive of both mortality and chronic infection with cytomegalovirus (CMV) in aging Swedish cohorts [4,7-9].

The nursing home elderly are a group that are at high risk of infection, vaccine failure and mortality, and it is postulated that immunosenescence is associated with this increased risk [10]. However, immune phenotypes in this group have not been well characterized, as most immunosenescence studies have excluded the nursing home elderly [10]. Three studies have investigated the immune phenotype of peripheral blood mononuclear cells (PBMCs) in the nursing home elderly [3,11,12]; two of these reports contained an elderly population composed of community dwelling and nursing home elderly and did not report specifically on the latter group [11,12], whereas the other study enrolled exclusively nursing home elderly [3]. The overall observations from these studies concluded that the nursing home elderly displayed immunosenescent phenotypes similar to the community dwelling elderly. However, it remains unclear whether immune phenotypes in the nursing home elderly are influenced by frailty and malnutrition; two conditions that are present in the nursing home elderly [13,14] and thought to influence immunosenescence [15-17]. Furthermore, the changes to a separate class of T-cell, the regulatory CD4+ T-cell (T-reg) and to the innate immune natural killer (NK) cell subsets have not yet been described in the nursing home elderly. The goal of this study therefore was to characterize T-cell and NK cell subsets within the nursing home elderly as a precursor to the identification of candidate immune biomarkers.

To this end, we examined circulating CD4+ and CD8+ T-cells and T-cell subsets (naïve, central memory, effector memory, terminally differentiated and senescent T-cells), T-regs and NK cell subset (immature, mature and senescent NK cells) in the nursing home elderly compared to healthy adults, and explored how individual immune phenotypes were influenced by age, sex, frailty and nutritional status in the nursing home elderly.

## Methods

### Subjects and setting

Elderly participants were recruited from four nursing homes in Hamilton, Ontario between September 2010 and December 2011. Nursing home residents ≥65 years were eligible. Residents on immunosuppressive medications (including cancer chemotherapy, oral corticosteroid use >21 days, methotrexate, post-transplant medications and/or anti-cytokine or B-lymphocyte depletion therapies) were excluded, as were participants affected by serious diseases with very poor short-term prognosis (as determined by the supervising physician). Written informed consent was obtained for all participants. A convenience sample of healthy adult participants (≥18 years and <65 years) were recruited from McMaster University in Hamilton, Ontario between September and December 2011. Healthy adults were laboratory students and staff who responded to an advertised request by the study investigators for participants. The study had Research Ethics Board approval from Hamilton Health Sciences and McMaster University Health Sciences ethics boards.

A research nurse abstracted baseline demographics from the nursing home elderly based on participant interview, examination and chart review. Frailty was rated according to the Clinical Frailty Scale, an 8-point scale ranging from very fit (1) to very severely frail (8), which has been validated in the nursing home population [13]. Nutritional status was assessed using the Mini Nutritional Assessment which categorized residents as having normal nutritional status, being at risk of malnutrition or malnourished [14].

### Whole blood analysis and flow cytometry

Whole blood was obtained from participants between 7 am and 10 am and hand delivered to the research laboratory for immediate processing. An aliquot of whole blood was used for phenotyping. T-cell phenotypes were determined by staining PBMCs in round-bottom 96-well plates with anti-CD3-Qdot605, anti-CD8-Alexa Flour 700, anti-CD4-Pacific Blue, anti-CD45RA-PE Texas Red, anti-CD28-PE, anti-CD57-FITC, anti-CCR7-PE Cy7. T-regs were identified using anti-CD3-FITC, anti-CD4-Pacific Blue, anti-CD127-PerCP-Cy5.5, anti-CD25-PE, and anti-FoxP3-AlexaFluor700. NK phenotypes were determined by staining PBMCs with anti-CD3-FITC, anti-CD56-PECy7 and anti-CD16-AlexaFluor700. The following antibodies were purchased from BD Bioscience: anti-CD4-Pacific Blue, anti-CD28-PE, anti-CCR7-PE-Cy7, anti-CD25-PE, anti-CD56-PE-Cy7, and anti-CD16-AlexaFluor700. The following antibodies were purchased from eBioscience: anti-CD3-FITC, anti-CD127-PerCP-Cy5.5, anti-FoxP3-AlexaFluor700. The anti-CD3-Qdot605 was purchased from Invitrogen. The anti-CD57-FITC and anti-CD45RA-PE-TexasRed antibodies were purchased from Beckman Coulter. We defined the T-cell subsets as follows: naïve (CD45 + CCR7+), central memory (CD45-CCR7+), effector memory (CD45-CCR7-), terminally differentiated (CD45 + CCR7-) and senescent (CD28-CD57+). T-regs were defined as CD4 + CD25hiCD127loFoxp3+. NK cells were determined based on CD16 and CD56 expression. CD56^bright^CD16- were classified as immature NK cells, CD56^dim^CD16+ were classified as mature NK cells, and a recently described NK cell subset, CD56^dim^CD16-, was classified as senescent NK cells [18]. CD4+ and CD8+ immune phenotypes, T-regs and NK cell subsets were expressed as a percentage of total lymphocytes. Antibody staining was performed using a Beckman Coulter Biomek NX^p^ Laboratory Automation Workstation (Beckman Coulter, Ontario) as described in our recent publication [19], followed by analysis using an LSR II flow cytometer with a high-throughput sampler (BD Biosciences, NJ, USA), and data was analyzed using FlowJo 9.6 (Treestar Inc, Ashland, OR).

PBMCs were isolated and frozen using a validated common standard operating procedure [20]. PBMCs were thawed and CMV-reactive T-cells were identified by stimulating PBMCs with a pool of overlapping peptides spanning the immunodominant pp65 protein of CMV (PepTivator pp65, Miltenyi Biotec) according to our published protocols [21]. Briefly, thawed PBMCs were cultured overnight at 37°C and stimulated with CMV peptides (2 ug/ml) for 1 hr at 37°C. A matched set of PBMCs were stimulated with DMSO as a negative control. Brefeldin A (BD Biosciences) was then added according to the manufacturer’s instructions and the cells were incubated for an additional 4 hours. The cells were stained with anti-CD4-PacificBlue and anti-CD8-AlexaFluor700, permeabilized and finally stained with anti-IFN-γ-APC, anti-TNF-α-FITC and anti-CD3-QDot605. CMV-reactive T-cells were identified as CD3+ (CD4+ or CD8+) IFN-γ + TNF-α + .

### Data analysis

Many of the immune phenotype distributions were skewed thus the results were summarized as medians and interquartile ranges (IQR). Non-parametric tests of significance were used including Mann–Whitney U, Kruskal-Wallis and Spearman’s rank correlation as appropriate. All statistics were performed using SPSS version 22.0 (SPSS Inc., Chicago, Illinois).

## Results

### Demographics

In total, 262 nursing home elderly were enrolled; ages ranged from 65 – 98 years, median age was 87 years (IQR 82–91) and 81% were female. The majority (94%) had at least one co-morbidity, and over half (52%) had a diagnosis of dementia (Table 1). Sixty percent of the participants scored 5 or 6 on the Clinical Frailty Scale, categorizing them as either mildly frail or moderately frail [13] (Table 1) and the median frailty according to the Clinical Frailty Scale was 6 (IQR 5–6), [13]. Sixteen healthy adults were enrolled; median age was 31 (IQR 27–36) and 50% were female (Table 1).

**Table 1:** Baseline characteristics of the nursing home elderly and healthy adults

**Baseline characteristics**	**Nursing home elderly n = 262**	**Healthy adults n = 16**
	**n (%)**	**n (%)**
Age (years)		
18-24	-	2 (13)
25-34	-	9 (56)
35-44	-	4 (25)
45-54	-	1 (6)
55-64	-	0 (0)
65-74	21 (8)	-
75-84	73 (28)	-
85-94	147 (56)	-
≥95	21 (8)	-
Sex (F)	213 (81)	8 (50)
Any co-morbidity	245 (94)	0 (0)
Diabetes	75 (29)	-
Stroke	56 (21)	-
Heart failure	36 (14)	-
Coronary artery disease	81 (31)	-
Cancer	21 (8)	-
COPD	30 (11)	-
Dementia	137 (52)	-
≥5 medications	229 (87)	0 (0)
Nutritional status		
At risk of malnutrition	121 (46)	0 (0)
Malnourished	13 (5)	0 (0)
Frailty		
4	64 (24)	0 (0)
5	62 (24)	0 (0)
6	94 (36)	0 (0)
7	38 (15)	0 (0)
8	4 (1.5)	0 (0)

### Description of immune phenotypes

There was no significant difference between the medians of CD8+ T-cell% in the nursing home elderly and healthy adults (median 17.1 versus 18.0, p = 0.56), however there was a significantly lower naïve CD8+ T-cell% median in the nursing home elderly compared to healthy adults (median 0.9 versus 5.2, p < 0.001), significantly higher terminally differentiated CD8+ T-cell% median in the nursing home elderly compared to healthy adults (median 7.3 versus 4.1, p = 0.004) and significantly higher CD8+ senescent T-cell% median in the nursing home elderly group when compared to the healthy adults (median 5.3 versus 3.1, p = 0.04) (Table 2).

**Table 2:** Immune phenotypes in the nursing home elderly compared to healthy adults

**Immune phenotype**	**Nursing home elderly**	**Healthy adults**	p-value*
	**Median (IQR)**	**Median (IQR)**	
	**n = 262**	**n = 16**	
**CD8+**			
CD8 + %	17.1 (11.9 – 23.9)	18.0 (15.1 – 21.0)	0.56
Naïve CD8 + %	0.90 (0.5 – 1.4)	5.2 (3.8 – 7.9)	<0.001
Central memory CD8 + %	0.6 (0.3 – 1.0)	0.5 (0.4 – 0.8)	0.86
Effector memory CD8 + %	6.3 (3.9 – 9.4)	8.1 (6.6 – 10.1)	0.14
Terminally differentiated CD8 + %	7.3 (3.5 – 12.5)	4.1 (2.0 – 6.0)	0.004
Senescent CD8 + %	5.3 (2.2 – 9.4)	3.1 (0.7 – 6.2)	0.04
**CD4+**			
CD4 + %	45.5 (38.0 - 52.7)	37.1 (29.7 - 43.1)	0.001
Naïve CD4 + %	9.2 (5.1 – 13.7)	11.6 (7.1 - 15.4)	0.19
Central memory CD4 + %	12.6 (9.1 - 16.2)	8.4 (6.5 - 13.2)	0.003
Effector memory CD4 + %	18.6 (14.1 - 22.6)	12.3 (11.8 - 15.8)	<0.001
Terminally differentiated CD4 + %	1.8 (1.1 – 2.8)	1.2 (0.6 - 2.1)	0.09
Senescent CD4 + %	1.4 (0.2 - 3.4)	0.8 (0.1-2.0)	0.21
**T-reg**			
T-reg%	1.8 (1.1 – 2.6)	0.8 (0.6 – 0.9)	<0.001
**Ratio**			
CD4 + : CD 8 + ratio	2.6 (1.7 – 4.1)	1.9 (1.6 – 2.6)	0.048
**NK**			
Mature NK cell%	12.1 (7.9 – 16.6)	5.4 (2.7 – 10.0)	0.001
Senescent NK cell%	1.4 (0.89 – 2.2)	1.7 (1.5 – 2.6)	0.27
Immature NK cell%	0.2 (0.1 – 0.4)	0.3 (0.04 – 0.7)	0.57

*Mann–Whitney U test of significance used.

There was a significantly higher CD4+ T-cell% median in the nursing home elderly compared to healthy adults (median 45.5 versus 37.1, p = 0.001) (Table 2). The overall increase in CD4 + % appeared due to higher central memory T-cell% and effector memory T-cell% in the nursing home elderly when compared to healthy adults (central memory% median 12.6 versus 8.4, p = 0.003 and effector memory% median 18.6 versus 12.3, p < 0.001) but not the terminally differentiated memory CD4+ T-cell% in the nursing home elderly when compared to healthy adults (median 1.8 versus 1.2, p = 0.09) (Table 2). In the nursing home elderly, females had higher median CD4 + % than males (median 46.1 versus 42.1, p = 0.02), higher median naïve CD4 T-cell% (median 10.1 versus 7.1, p = 0.04) and higher median terminally differentiated CD4+ T-cell% (median 1.9 versus 1.4, p = 0.03) (Table 3).

**Table 3:** Immune phenotypes as a function of age, sex, frailty and nutritional status

**Immune phenotype**	**Age Correlation**^**+ **^**(r) n = 262**	**Sex Median (IQR **) **n = 262**^**++**^		**Clinical Frailty Scale Median (IQR) n = 262**^**+++**^				**Nutritional Status Median (IQR) n = 261**^**a+++**^		
		**Female n = 213**	**Male n = 49**	**4 n = 64**	**5 n = 62**	**6 n = 94**	**7 or 8 n = 42**	**Normal n = 127**	**At risk n = 121**	**Mal-nourished n = 13**
CD8+										
CD8 + %	0.05	17.1	17.4	18.6	17.6	16.3	16.3	17.4	17.1	17.1
		(11.8-24.2)	(12.8-22.2)	(13.7-23.8)	(12.3-21.9)	(11.7-24.1)	(9.7-26.8)	(12.2-23.8)	(11.7-24.2)	(11.2-20.9)
Naïve CD8 + %	−0.13*	0.9	0.8	0.9	0.8	1.0	1.2	0.9	1.0	0.7
		(0.5-1.5)	(0.4-1.2)	(0.5-1.4)	(0.5-1.4)	(0.5-1.4)	(0.5-2.1)	(0.5-1.4)	(0.58-1.6)	(0.3-1.2)
Central memory CD8 + %	−0.03	0.6	0.5	0.5	0.5	0.6	0.7	0.5	0.7	0.5
		(0.3-1.0)	(0.3-0.8)	(0.3-0.9)	(0.3-0.9)	(0.3-1.0)	(0.3-1.5)	(0.3-0.9)	(0.3-1.1)	(0.4-0.9)
Effector memory CD8 + %	−0.04	6.1	7.1	6.7	6.3	6.3	5.7	6.5	6.0	6.0
		(3.8-9.5)	(4.5-9.7)	(4.1-9.9)	(4.1-8.7)	(3.7-10.0)	(4.0-8.6)	(3.8-9.9)	(3.9-8.8)	(4.0-10.9)
Terminally differentiated CD8 + %	0.08	7.8	5.9	8.9	7.6	6.9	6.1	8.1	7.2	5.8
		(3.6-12.6)	(3.3-12.5)	(4.2-13.3)	(3.7-12.8)	(3.4-11.0)	(2.4-12.9)	(3.5-12.7)	(3.6-12.5)	(3.1-10.9)
Senescent CD8 + %	0.04	5.1	5.7	6.2	5.0	5.1	4.9	5.1	5.2	5.6
		(2.3-8.9)	(2.0-11.3)	(2.8-11.4)	(2.3-9.3)	(2.2-7.9)	(0.8-10.0)	(2.2-10.0)	(2.3-8.6)	(1.2-9.5)
CD4+										
CD4 + %	−0.06	46.1*	42.1*	44.9	45.8	46.4	43.1	46.1	45.8	42.0
		(39.1-53.8)	(34.7-49.1)	(38.6-53.6)	(37.1-52.6)	(40.4-53.1)	(32.4-52.7)	(38.7-52.6)	(36.1-53.5)	(35.9-44.9)
Naïve CD4 + %	0.05	10.1*	7.1*	10.5	9.2	9.3	7.2	10.4*	8.7*	5.9*
		(5.4-14.6)	(4.6-11.5)	(5.4-18.0)	(5.3-14.7)	(5.4-13.1)	(4.0-12.2)	(5.4-16.1)	(4.8-12.5)	(3.4-9.9)
Central memory CD4 + %	−0.08	12.8	11.7	12.5	12.0	13.3	12.8	12.5	13.1	10.7
		(9.6-16.4)	(8.7-14.6)	(9.0-15.4)	(8.4-16.2)	(10.7-18.2)	(8.7-15.8)	(9.2-16.2)	(9.0-17.2)	(9.3-13.3)
Effector memory CD4 + %	0.13*	18.9	18.2	19.3	18.1	19.2	17.0	18.6	19.0	18.5
		(14.6-23.0)	(12.9-21.4)	(13.6-21.8)	(12.7-21.9)	(15.2-24.2)	(13.5-22.4)	(13.5-21.8)	(14.6-22.8)	(12.8-26.6)
Terminally differentiated CD4 + %	0.05	1.9*	1.4*	1.9	1.8	1.6	1.8	1.9	1.6	1.4
		(1.1-2.9)	(0.6-2.5)	(1.1-3.3)	(1.1-2.6)	(1.1-2.7)	(0.8-2.7)	(1.1-2.9)	(1.0-2.8)	(0.9-2.0)
Senescent CD4 + %	0.11	1.4	1.3	1.5	1.4	1.4	1.2	1.4	1.3	2.0
		(0.3-3.5)	(0.2-2.9)	(0.3-4.2)	(0.1-3.1)	(0.3-3.6)	(0.2-2.9)	(0.16-3.4)	(0.27-3.2)	(0.34-4.4)
T-reg										
T-reg%	0.08	1.8	1.6	2.5 **	2.0**	1.6**	1.0**	2.2**	1.6**	1.0**
		(1.2-2.6)	(0.9-2.6)	(1.8-2.9)	(1.3-2.6)	(1.1-2.3)	(0.8-1.5)	(1.5-2.9)	(1.1-2.3)	(0.8-1.2)
Ratio										
CD4 + : CD 8 + ratio	−0.06	2.6	2.5	2.5	2.4	2.7	2.6	2.6	2.6	2.3
		(1.7-4.5)	(1.6-3.5)	(1.6-3.8)	(1.8-4.1)	(1.9-4.5)	(1.4-5.0)	(1.7-4.1)	(1.6-4.5)	(1.8-3.3)
NK										
Mature NK cell%	−0.05	12	12.6	12.0	11.3	12.3	12.7	12.3	11.6	14.7
		(8.0-16.6)	(7.1-17)	(8.3-6.3)	(7.1-15.2)	(7.3-16.7)	(8.5-17.2)	(8.7-16.0)	(6.8-16.5)	(7.9-18.4)
Senescent NK cell%	−0.10	1.4	1.5	1.0**	1.1**	1.7**	2.0**	1.3*	1.6*	1.4*
		(0.9-2.2)	(1.0-2.4)	(0.7-1.9)	(0.7-1.9)	(1.0-2.7)	(1.3-2.7)	(0.74-2.1)	(1.0-2.7)	(1.1-2.3)
Immature NK cell%	0.03	0.2	0.3	0.2	0.21	0.3	0.3	0.2	0.2	0.2
		(0.1-0.4)	(0.1-0.5)	(0.1-0.4)	(0.1-0.3)	(0.2-0.4)	(0.2-0.4)	(0.2-0.4)	(0.1-0.4)	(0.13-0.45)

*p ≤ 0.05.

**p ≤ 0.001.

^a^One patient excluded as nutritional status unknown.

^+^Spearman’s rank test of significance used.

^++^Mann–Whitney U test of significance used.

^+++^Kruskal-Wallis test of significance used.

There was significantly higher median T-reg% in the nursing home elderly group when compared to the healthy adults (median 1.8 versus 0.8, p < 0.001) (Table 2). In the nursing home elderly, there were significant differences in median T-regs% across frailty (p < 0.001) and nutritional status (p < 0.001) (Table 3).

When the CD4+/CD8+ T-cell ratios were compared, the nursing home elderly had a higher CD4+/CD8+ T-cell ratio than the healthy adults (median CD4+/CD8+ ratio 2.6 versus 1.9, p = 0.048) (Table 2). In the nursing home elderly, the CD4+/CD8+ ratio ranged from 0.42 – 22 and only 17 (6.5%) had a CD4+/CD8+ ratio <1.0. This was in contrast to a far narrower range of values in the healthy adults (CD4+/CD8+ ratio range 1.1 – 3.5). In the nursing home elderly, age, sex, frailty and nutritional status were not associated with changes to the CD4+/CD8+ ratio (Table 3).

There was an increase in the mature NK cell% in the nursing home elderly compared to the healthy adults (median 12.1 versus 5.4, p = 0.001) and no significant difference between senescent NK cell% (median 1.4 versus 1.7, p = 0.27) or immature NK cell% (median 0.2 versus 0.3, p = 0.57) (Table 2). In the nursing home elderly, higher senescent NK cell% was associated with increasing frailty (p < 0.001) and worsening nutrition (p = 0.01) (Table 3). Age, sex, frailty and nutrition did not appear to have a significant association with mature or immature NK cells (Table 3).

Of the 262 nursing home elderly enrolled in the study, 242 had PBMCs available for assessment of T-cell immunity to CMV. In total, 217/242 (90%) of individuals had evidence of prior CMV infection.

## Discussion

In this crosssectional study in the nursing home elderly, we observed 1) lower naïve CD8+ T-cells and higher terminally differentiated and senescent CD8+ T-cells; 2) higher CD4+ T-cells driven by central and effector memory CD4+ T-cells; 3) higher CD4+/CD8+ T-cell ratio; 4) higher T-regs; and 5) higher mature NK cells and when compared to healthy adults.

The lower naïve CD8+ T-cells and higher terminally differentiated CD8+ T-cells seen in this study in the nursing home elderly has been well described in community dwelling elderly [2,3,22]. These differences are consistent with existing dogma, that the remodelling of the T-cell compartment seen with advanced age is due to a decrease in naïve T-cells due to thymic involution and an increase in terminally differentiated T-cells, possibly due to chronic antigenic stimulation from CMV [1]. Indeed, 90% of the elderly nursing home residents in our study had evidence of CMV immunity, consistent with prior studies where infection with CMV is estimated to be prevalent in >85% of those aged over 80 years [23]. The reduction of naïve T-cells is thought to impair the ability of the host to respond against novel pathogens [10,24] and the terminally differentiated memory T-cells are considered to have poor functionality resulting in impaired responses to recall antigens [24], supporting the hypothesis that these immune changes increase the risk of infection and mortality in the elderly.

Higher senescent CD8 + CD28- T-cells have been associated with advancing age [3,4], influenza vaccine failure [5,6] and CMV infection [4]. In this study, there was a higher median senescent CD8+ T-cells in the nursing home elderly when compared to healthy adults. The one prior immunosenescence study performed in the nursing home elderly also reported higher senescent T-cells when compared to healthy adults [3].

The described differences in CD4+ T-cells in the elderly have been far more inconsistent than for CD8+ T-cells. Studies have described both higher CD4+ T-cells [25,26] and lower CD4+ T-cells [2,4,9,27,28]. Two studies found lower CD4+ T-cells in malnourished elderly [16,17] and the one study that enrolled exclusively nursing home residents showed no change at all [3]. It is possible, that the higher CD4 + % seen in our study is partially explained by the predominance of females in the nursing home group (81%), as females had higher CD4% than men, a finding consistent with the literature [25]. The high proportion of females enrolled in our study in the nursing home elderly group was expected given that nursing homes in Canada are comprised of approximately 70% females [29]. Our CD4 + T-cell subset results, that the higher circulating CD4+ T-cells in the nursing home elderly was due to higher central memory and effector memory T-cells, are consistent with another immunosenescence study that enrolled community elderly and used newer immune phenotyping better able to delineate the naïve and memory T-cells pools [2].

The expanded CD4+ T-cell compartment in the nursing home elderly in our study is likely why the CD4+/CD8+ T-cell ratio was higher in the nursing home elderly group than the healthy elderly and only 6% of the nursing home elderly had a CD4+/CD8+ ratio <1.0. This is in contrast to the Swedish OCTO (n = 102) and NONA (n = 138) cohorts where 14% and 20% of the cohorts had CD4+/CD8+ ratios <1.0 [4,7]. Our results are very similar to another study that included exclusively nursing home elderly (n = 116); the CD4+/CD8+ ratio was 2.0 in the elderly and 1.7 in the young cohort (the difference was not significant, p = 0.64) [3]. In a Spanish study that enrolled 151 elderly people ≥65 years only 7.9% of the cohort had a CD4+/CD8+ T-cell ratio <1.0 [30]. The reason for the discrepancy in results among the studies is not clear, but could relate to differences in population. First, only 34% in the NONA cohort were in institutional care [4] versus 100% in our study. Second, the proportion of females enrolled could influence the CD4+/CD8+ ratio given the noted association between female sex and CD4 + %. Both our study and the other study that included exclusively nursing home elderly were comprised of approximately 80% females [3] whereas the OCTO and NONA studies consisted of 65% and 70% females, respectively [31]. Last, the OCTO and NONA studies were conducted in Sweden whereas this and the other nursing home study are North American [3]. Indeed, 8% of younger adults in Sweden (ages 20–59) have a CD4+/CD8+ T-cell ratio <1.0 [31]. The NONA cohort has also demonstrated that at very advanced ages (>90 years), it is possible to move from having a CD4+/CD8+ T-cell ratio <1.0 to above 1.0, which is another possible reason why our study had so few people with CD4+/CD8+ T-cell ratios <1.0 [8]. Our findings raise the possibility that CD4+/CD8+ ratios <1.0 may not be predictive of increased mortality in the nursing home elderly, particularly given that this ratio tended to increase in residents with severe frailty.

The accumulation of T-regs has been consistently observed in studies of aging involving the community elderly [32]. How higher T-regs could lead to impairment of host control of infection is not clear, but may be secondary to a decreased ability of aged T-regs to proliferate and produce cytokines [33]. It is interesting to note that the median T-regs% was lower in healthy adults when compared to nursing home elderly, but also in the very frail and the malnourished nursing home elderly. To our knowledge, no other study has reported on the relationship between T-regs and frailty or nutrition. Further study into the relationship between T-regs and human immunosenescence is required.

The higher mature NK subset seen in the nursing home elderly in our study is consistent with immunosenescence studies including community dwelling elderly [34,35]. These results support the observation that the increase in NK cells seen in the elderly is due to accumulation of mature NK cells. We speculate that the higher numbers of mature NK cells could be due to the same mechanism leading to higher numbers of memory T-cells in the elderly; chronic antigenic stimulation [1]. Although the medians of the recently described senescent NK cells [18] did not differ between the nursing home elderly and the healthy adults, there was higher senescent NK cell medians in those with advancing frailty and worsening nutrition.

Although our study has many strengths including a large sample size of nursing home residents and detailed clinical information including frailty and nutritional status as well as comprehensive immune phenotyping, limitations of our study include the small size of the comparator group, which may have increased our risk of not finding a difference between the two groups when one exists (type II error). However our control group size was comparable to at least four other similar studies where the control group size ranged from 15 to 21 adults [3,8,36,37] and our findings were similar to studies involving community dwelling elderly, increasing the confidence in our results. In addition, due to our study design, we are unable to conclude that the observed differences in immune phenotypes between the nursing home elderly and healthy adults are necessarily associated with aging. However, the goal of this study was not to determine associations, but to characterize differences in T-cell and NK cell subsets in the nursing home elderly, to aid in the identification of immune biomarkers in future studies involving the nursing home elderly.

## Conclusion

Differences in naïve CD8+ T-cells, terminally differentiated CD8+ T-cells, T-regs and NK cells subsets in the nursing home elderly were similar to studies involving community dwelling elderly. However, the CD4+/CD8+ T-cell ratio was higher in our study population. These results will aid future studies designed to investigate immune biomarkers predictive of infection, vaccine failure and mortality in the nursing home elderly.

## Competing interests

The authors declare that they have no competing interest.

## Authors’ contributions

JJ, ML and JB were responsible for the conception and design of the study, acquisition of data, analysis and interpretation of data. JM, AL, CV, SW and PD aided in data analysis and interpretation of data. JJ drafted the initial manuscript and JM, AL, CV, SW, PD, JB and ML critically revised the manuscript for intellectual content. All authors read and approved the final version of the manuscript.

## Authors’ information

Jonathan Bramson and Mark Loeb are co-senior authors.

## Pre-publication history

The pre-publication history for this paper can be accessed here:

http://www.biomedcentral.com/1471-2318/14/50/prepub
